# Enhanced IL-10 production in response to hepatitis C virus proteins by peripheral blood mononuclear cells from human immunodeficiency virus-monoinfected individuals

**DOI:** 10.1186/1471-2172-9-28

**Published:** 2008-06-13

**Authors:** Lisa Barrett, Maureen Gallant, Constance Howley, M Ian Bowmer, Geri Hirsch, Kevork Peltekian, Michael Grant

**Affiliations:** 1 Immunology and Infectious Diseases Program, Division of BioMedical Sciences, Faculty of Medicine, Memorial University, St. John's, Canada; 2 HIV Program, Eastern Health District, St. John's, Canada; 3 Hepatitis C Program, Division of Gastroenterology, Capital Health District, Halifax, Canada; 4 Department of Internal Medicine, Dalhousie University, Halifax, Canada

## Abstract

**Background:**

Multiple immune evasion strategies by which HCV establishes chronic infection have been proposed, including manipulation of cytokine responses. Prior infection with HIV increases the likelihood of chronic HCV infection and accelerates development of HCV-related morbidity. Therefore, we investigated in vitro cytokine responses to HCV structural and non-structural proteins in peripheral blood mononuclear cells (PBMC) from uninfected, HIV-infected, HCV-infected and HIV/HCV-coinfected individuals.

**Results:**

Intracellular flow cytometry was used to assess IL-2, IL-10, IL-12, and IFN-γ production by freshly isolated PBMC incubated for 16 hours with recombinant HCV core, non-structural protein 3 (NS3), and NS4 proteins. Anti-HCV cellular responses were assessed in HIV/HCV-coinfected individuals by ^3^H-thymidine proliferation assay. Exposure to HCV antigens increased IL-10 production by PBMC, especially in uninfected and HIV-monoinfected individuals. This IL-10 response was attenuated in chronic HCV infection even with HCV/HIV-coinfection. The cells producing IL-10 in response to HCV proteins in vitro matched a PBMC subset recently shown to constitutively produce IL-10 in vivo. This subset was found at similar frequencies in uninfected, HIV-infected, HCV-infected and HIV/HCV-coinfected individuals before exposure to HCV proteins. HCV-specific T cell proliferation was detectable in only one HIV/HCV-coinfected individual who demonstrated no HCV-induced IL-10 response.

**Conclusion:**

This pattern suggests that selective induction of IL-10 in uninfected individuals and especially in HIV-monoinfected individuals plays a role in establishing chronic HCV infection and conversely, that attenuation of this response, once chronic infection is established, favours development of hepatic immunopathology.

## Background

Most viral infections induce cellular and humoral immune responses that act in concert to limit viral spread, clear infection and provide protective immunity against reinfection with the same virus. However, a number of viruses have evolved varied and sophisticated mechanisms to establish persistent infection, even in immunocompetent hosts [1-4]. Epstein-Barr virus (EBV) produces an interleukin-10-like virokine that functionally mimics IL-10 in downregulating cellular immune responses [4].

Hepatitis C virus (HCV) causes significant morbidity and mortality. Approximately 80% of HCV-exposed individuals develop life-long infection and in many cases, progressive liver disease [5]. Immune escape through mutations introduced by the error prone HCV RNA-dependent RNA polymerase is believed to play a major role in the establishment of persistent HCV infection as strong, broadly directed cellular responses during acute infection are associated with HCV clearance, while narrow, qualitatively impaired anti-HCV responses occur in chronic infection [6-12]. Clearance of HCV after interferon-alpha (IFN-α) and ribavirin therapy is also associated with greater breadth and amplitude of anti-HCV cellular immunity [13,14]. These associations suggest that development of appropriate adaptive immune responses relates closely to HCV clearance.

The relatively small RNA genome of HCV encodes few proteins, all of which are involved in some way with virion structure or virus replication. Hence, any immunosuppressive activity must reflect secondary functions of the structural or non-structural HCV proteins. Core, non-structural protein 3 (NS3), NS4 and NS5 have been investigated for a variety of secondary functions. The 20 kDa HCV core protein is associated with cellular transformation in vitro and modulation of cytokine signalling and cellular immune responses [15-17]. The NS3 serine protease downregulates immunoproteosome activity in vitro and may impair immune recognition [18]. NS4 is comprised of NS4A and NS4B, two proteins involved in viral replication. NS4A acts as a cofactor in forming the active protease NS4A/NS3 that participates in polyprotein processing during HCV replication [19], while NS4B is essential for replication and seems to affect the intracellular membranous web where viral replication occurs [20]. NS5A inhibits antiviral PKR activity through its interferon sensitivity determining region (ISDR) [21]. Thus, multiple HCV proteins appear to have evolved secondary functions affecting both innate and adaptive immune responses.

Viral infection generally triggers production of pro-inflammatory cytokines, but as described above, viruses have evolved gene products that promote persistent infection by tipping the cytokine balance towards immunosuppression. The setting in which viral exposure initially occurs is also relevant, therefore, we compared induction of cytokines by HCV proteins in PBMC from uninfected, HCV-infected, HIV-infected and HIV/HCV-coinfected individuals. Production of the immunosuppressive cytokine IL-10 was selectively triggered by HCV proteins, and the response was greater in HIV-monoinfected and uninfected individuals than in those exposed to HCV. This pattern suggests that selective induction of IL-10 in uninfected individuals and especially in HIV-monoinfected individuals plays a role in establishing chronic HCV infection and conversely, that attenuation of this response, once chronic infection is established, favours development of hepatic immunopathology.

## Results

### Study participants

Eight healthy volunteers from St. John's NL, Canada provided blood samples for the study. Twenty-four HIV-infected, 10 HIV/HCV-coinfected, and 3 HCV-infected individuals were also recruited, and Table 1 describes the baseline characteristics of the infected groups. The age and sex of participants was similar across groups. Most individuals exposed to HCV developed chronic infection, although 2 of the HIV/HCV-coinfected individuals had cleared the virus and were anti-HCV antibody positive but HCV RNA negative. HIV related clinical parameters were similar between groups, although there was a statistically nonsignificant trend toward higher CD4^+ ^T cell counts in the coinfected group. Most HIV-infected individuals were on antiviral therapy and had achieved viral suppression at the time of participation. Only one of the HIV/HCV-coinfected individuals had been treated (unsuccessfully) with HCV antiviral therapy, and the remainder of HCV exposed patients were untreated.

**Table 1:** Patient characteristics.

	**HIV**	**HIV/HCV**	**Chronic HCV**
**Number (n)**	24	10	3
**Age (yrs ± SD)**	41.7 ± 7.0	39.2 ± 3.85	49 ± 15
**Sex (%)**			
M	75	80	66
F	25	20	33
**HCV Genotype (%)**			
1	N/A	100	0
Non-1	N/A	0	0
Unknown	N/A	0	100
**HCV status (%)**			
Chronic infection (HCV RNA +)	N/A	80	100
Clearance (HCV RNA -)	N/A	20	0
**HCV Risk Factor (%)**			
IVDU	N/A	30	66.7
Transfusion	N/A	20	33.3
Tattoo	N/A	0	0
Endemic area	N/A	0	0
Unknown	N/A	40	0
**Estimated duration of infection (yrs ± SD)^1^**			
HIV	18 ± 5.1	10 ± 2.1	N/A
HCV	N/A	18 ± 3.4	24 ± 8
**Antiviral therapy (%)**			
HIV	58	90	N/A
HCV	N/A	0	0
**Suppressed viral load**^2^**(%)**			
HIV	75	70	N/A
HCV	N/A	20%	Unk.
**CD4^+ ^T cell count^3 ^**(median cells/μL, interquartile range)	409, 422	608, 80	N/A
**CD4^+ ^T cell nadir^4 ^**(median cells/μL, interquartile range)	252, 296	247, 350	N/A

Abbreviations: Avg average; N/A not applicable; SD standard deviation. ^1^The time since clinically confirmed infection or, in the case of HCV, high risk activity. ^2^Undetectable viral load by Roche Amplicor RT-PCR test for HIV and in house PCR for HCV at the time of participation. This also includes those who have spontaneously cleared the virus in the case of HCV. ^3^Measured on the day of participation. ^4^Lowest documented count over the course of followup.

### Exposure to HCV proteins induces IL-10 production in PBMC

**Figure 1:**
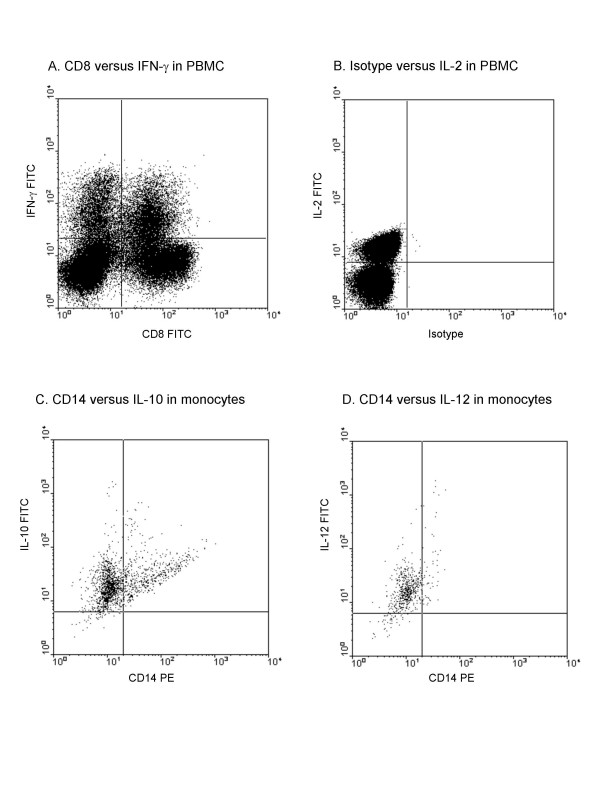
**Positive controls for cytokine production**. (a) IFN-γ production by CD8^+ ^and CD8^- ^lymphoid gated PBMC after phytohemagglutinin treatment. (b) IL-2 production in total PBMC after PMA/ionomycin treatment. (c) IL-10 production in CD14^+ ^cells after LPS stimulation. (d) IL-12 production in CD14^+ ^cells after LPS stimulation.

**Figure 2:**
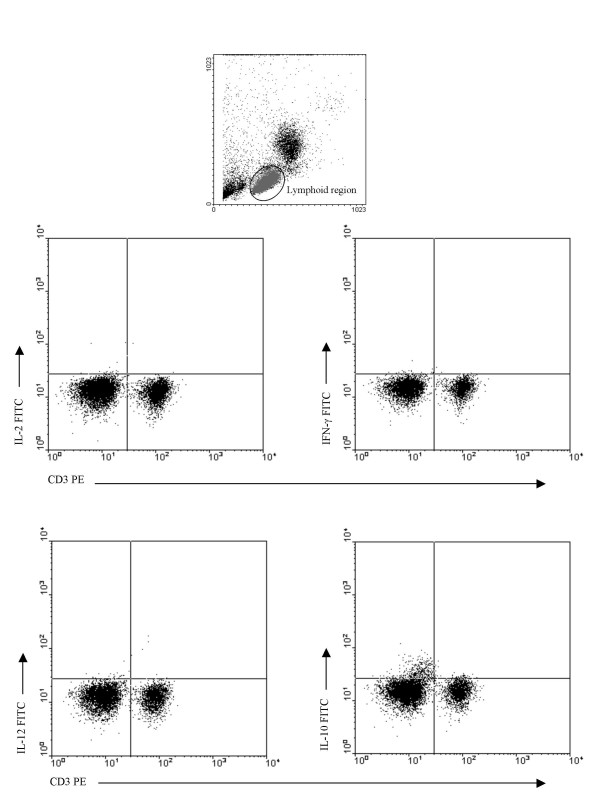
**Cytokine production following 16 hour exposure of PBMC to HCV proteins**. Freshly isolated PBMC from HIV-infected, HCV-infected, HIV/HCV co-infected and uninfected controls were incubated for 16 hours with HCV proteins, then analyzed by intracellular flow cytometry for cytokine production as shown in representative plots. Selective IL-10 production by a small subset of CD3^- ^PBMC with lymphoid scatter characteristics occurred in each case.

**Figure 3:**
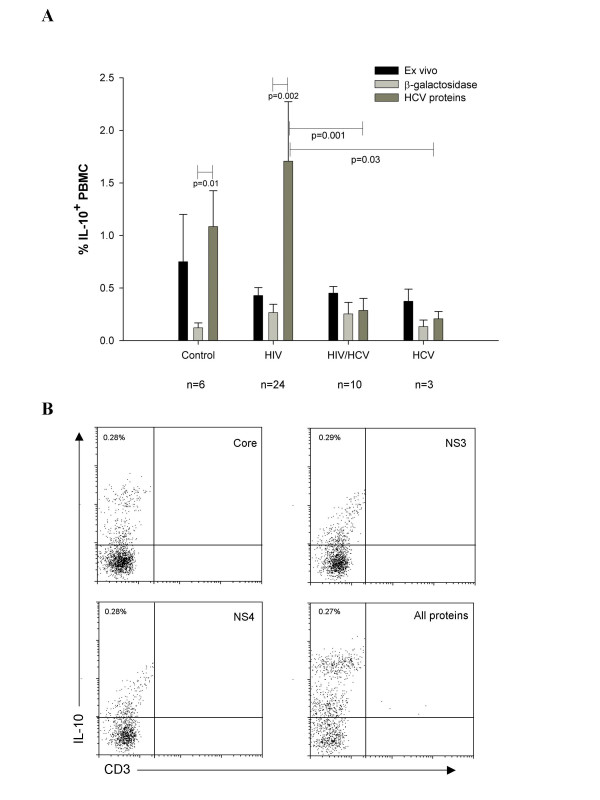
**Mean percentage of PBMC from different groups expressing IL-10 ex vivo and following 16 hour incubation with HCV proteins**. (a) The percentage of IL-10 producing cells (mean ± SE) was assessed by intracellular flow cytometry in freshly isolated PBMC and following 16 hour incubation with HCV proteins. Black bars represent fresh PBMC, dark grey bars represent PBMC incubated for 16 hours with recombinant HCV core, NS3 and NS4 and lighter grey bars represent the same PBMC incubated for 16 hours with β-galactosidase control protein. Statistical comparisons are made within groups using the Mann-Whitney U test and between groups using the Kruskal-Wallis test. (b) Representative flow plots of IL-10 production in PBMC from an HIV/HCV-coinfected individual after incubation of PBMC with individual HCV proteins at 2.5 μg/mL. Numbers represent the percentage of IL-10 positive PBMC.

To investigate the cytokine response of PBMC from different groups of individuals to HCV proteins, we incubated PBMC from uninfected, HIV-infected, HCV-infected and HIV/HCV-coinfected individuals for 16 hours with HCV core, NS3 and NS4 proteins. Production of IL-2, IL-12, IFN-γ and IL-10 was then assessed by intracellular flow cytometry. Positive controls for each of these four cytokines demonstrated the efficacy of the antibodies (Figure 1). In no case did any of the HCV antigens, individually or together, induce IL-2, IL-10, IFN-γ, or IL-12 production (Figure 2). However, IL-10^+^CD3^-^CD19^-^CD14^-^CD36^+^CD61^+^cells with lymphoid light scatter characteristics increased in number following for 16 hours exposure to HCV proteins (Figure 3a, grey bars). We previously described these IL-10^+ ^cells in freshly-isolated PBMC from uninfected individuals, but found that their number fell to almost undetectable levels following 16 hour incubation in unsupplemented lymphocyte medium [22]. All of the individuals in this study (uninfected n = 6, HIV-infected n = 24, HIV/HCV-coinfected n = 10, HCV mono-infected n = 3) had circulating CD36^+^CD61^+ ^PBMC constitutively producing IL-10 (Figure 3a, black bars). The average percentage of PBMC producing IL-10 ex vivo ranged from 0.37% (range 0.22–0.6%) in the HCV-infected group to 0.75% (range 0.2–1.2%) in the uninfected group. Although there was a trend towards a lower frequency of these cells in the HIV-infected, HCV-infected and HIV/HCV-coinfected groups, there was no statistically significant difference (Figure 3a). As previously described, in the absence of stimulation, the percentage of PBMC producing IL-10 in vitro decreased over time in infected and uninfected individuals (Figure 3a, light grey bars). The uninfected group had the highest frequency of IL-10 producing cells ex vivo (Figure 3a, black bars), however, after for 16 hours incubation in lymphocyte medium alone, IL-10^+ ^cell frequency fell to similar levels in all groups (Figure 3a, light grey bars). The mean frequencies were slightly lower in the uninfected and HCV-infected groups compared to the HIV-infected and HIV/HCV-coinfected groups (Figure 3a, light grey bars, 0.12% and 0.12% versus 0.25%, and 0.24% respectively) but not significantly different. The pattern of IL-10 production in response to HCV proteins differed substantially between groups (Figure 3a, dark grey bars). After 16 hour exposure to HCV proteins, the average frequency of IL-10^+ ^cells rose to 1.63% (n = 12; range 0.1% to 6.6%) in PBMC from HIV-infected individuals, 1.02% (n = 8; range 0.02% to 3%) in uninfected individuals, 0.27% (n = 12; range 0.01% to 1.56%) in HIV/HCV-coinfected individuals, and 0.18% (n = 3; range 0.06% to 0.26%) in HCV-infected individuals. The number of IL-10^+ ^cells detected by flow cytometry following 16 hour exposure to the HCV proteins was significantly higher in the HIV-infected group compared to the HCV-infected (H = 33, p = 0.03) and HIV/HCV coinfected (H = 15.5, p = 0.001) groups (Figure 3a). The effect of 16 hour exposure to HCV proteins on the number of IL-10^+ ^PBMC was quite substantial (5–10 fold increase) in HIV-infected and uninfected control groups, while the increase in HCV-infected individuals, with or without HIV coinfection, was marginal (Figure 3a). If all the chronic HCV-infected individuals from the HIV/HCV and HCV monoinfected groups are combined into one group (n = 13), there is still a significant difference between IL-10 induction in the HIV monoinfected group and the combined HCV chronic infection group (p = 0.016). IL-10 induction when HCV proteins were added together or individually was not different (Figure 3b).

**Figure 4:**
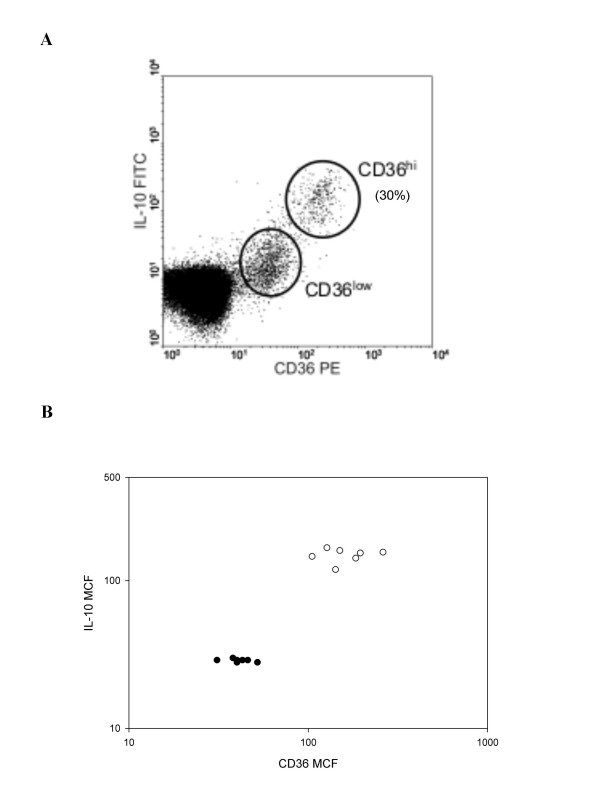
**Relationship between CD36 expression and ex vivo IL-10 production by PBMC**. (a) Representative flow cytometry plot showing IL-10 production in freshly-isolated CD36^+ ^PBMC cells with lymphoid light scatter characteristics. The percentage of CD36^+ ^cells that are IL-10^hi ^is indicated. (b) Mean channel fluorescence (MCF) for CD36 plotted against IL-10 MCF for six individuals. Open circles represent the CD36^hi ^subset and dark circles represent the CD36^low ^subset as shown in fig. 3a.

There were two clear populations of IL-10 positive cells based on the level of CD36 expression. Approximately 30% of the IL-10 positive cells had a CD36 mean channel fluorescence (MCF) greater than 100, and the remainder were between 10 and 100 (Figure 4a). Cells with higher CD36 mean channel fluorescence (MCF) were more likely to be IL-10 positive, with 100% of CD36^hi ^cells IL-10^+ ^versus 50–70% CD36^low ^cells IL-10^+ ^(Figure 4a). There is a statistically significant difference in the mean channel fluorescence of the CD36^lo^IL-10^lo ^group compared to the CD36^hi^IL-10^hi ^group (Figure 4b; Fisher's exact test, p < 0.000). This association raises the possibility of a mechanistic link between high CD36 expression and IL-10 production.

### IL-10 induction is specific to HCV proteins, and independent of T cells and lysosomal antigen processing

**Figure 5:**
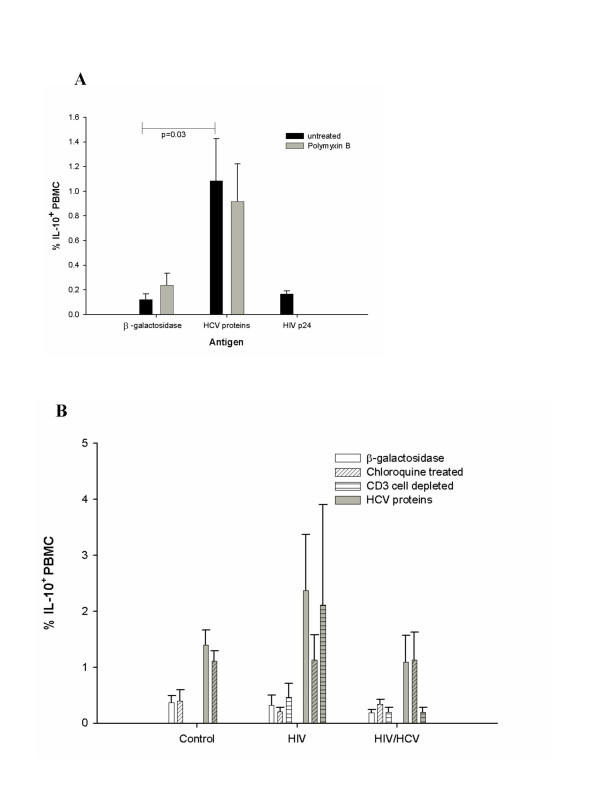
**Effect of polymyxin B, chloroquine and T cell depletion on IL-10 production by PBMC exposed to recombinant HCV proteins**. (a) Cells from uninfected individuals (n = 5) were pretreated with polymyxin B (grey bars) before incubation with recombinant HCV proteins to block the effects of any endotoxin contamination of the recombinant proteins. The effect of 16 hour exposure to recombinant HIV p24 on IL-10 induction was also assessed. (b) The mean percentage PBMC expressing IL-10 (± SE) from control, HIV-infected and HIV/HCV coinfected individuals was assessed after exposure to either β-galactosidase (white background bars) or HCV proteins (grey background bars). Effects of treating PBMC with chloroquine (diagonal hatch) or depleting CD3^+ ^T cells (horizontal hatch) are also shown for comparison with the HCV protein group (grey bars). Each individual was tested on at least 2 different visits.

To determine whether induction of IL-10 was selective for HCV proteins, we also exposed PBMC from 5 uninfected and 5 HIV-infected individuals to HIV p24 protein. Unlike the HCV proteins, HIV p24 did not increase IL-10 production in PBMC relative to the β-galactosidase controls (Figure 5a, black bars). Therefore, HCV core, NS3 and NS4 selectively induce IL-10 production in PBMC. To confirm that IL-10 induction did not result from endotoxin contamination of the HCV antigen preparations, we incubated PBMC from 5 uninfected and 3 HIV/HCV coinfected individuals with HCV proteins in the presence of polymixin B, an endotoxin blocker. Inclusion of polymixin B in the assays had no effect on induction of IL-10 in HCV-exposed PBMC (Figure 5a, light grey bars). To test whether antigen processing of the HCV proteins was required for induction of IL-10, we inhibited the lysosomal antigen processing pathway with chloroquine in PBMC from uninfected, HIV-infected and HIV/HCV-coinfected individuals exposed to HCV proteins (n = 3, n = 4, n = 4 respectively). Chloroquine treatment did not significantly affect the IL-10^+ ^cell frequency in PBMC from uninfected or HIV/HCV-coinfected groups exposed to HCV proteins (Figure 5b, grey bars with diagonal hatch).

Since the HCV proteins had the greatest effect on IL-10 induction in PBMC from HIV-infected individuals and reduced CD4^+ ^and total T cell numbers is an integral aspect of HIV infection, we decided to test if the absence of T cells affected the IL-10 response to HCV proteins. Therefore, we depleted CD3^+ ^T cells from the PBMC of 2 individuals in each of the HIV-infected and HIV/HCV-coinfected groups (Figure 5b, horizontal hatch bars) and assessed IL-10 production with and without exposure to HCV proteins (white or grey background bars respectively). The percentage of PBMC producing IL-10 constitutively or in response to HCV proteins was essentially unaffected by T cell depletion after correction for T cell depletion.

### Relationship between IL-10 induction and HCV-specific T cell proliferation

**Figure 6:**
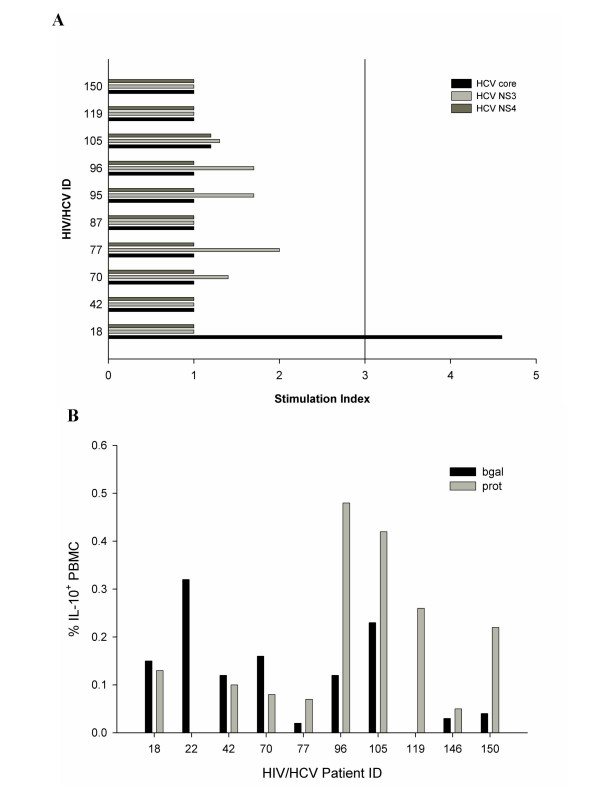
**Anti-HCV cellular responses and IL-10 induction in HIV/HCV coinfected Individuals**. (a) ^3^H-thymidine incorporation by PBMC from 10 HIV/HCV coinfected individuals incubated for 5 days with HCV core (black bar), NS3 (medium grey bar), NS4 (dark grey bar) or HIV p24 (light grey bar) was measured. A stimulation index of ≥3 was considered positive. Individuals 18 and 150 were anti-HCV positive but HCV RNA negative at the time of testing. (b) IL-10 production in 10 HIV/HCV coinfected individuals after 16 hour incubation with either β-galactosidase or HCV proteins. The percentage of IL-10^+ ^PBMC within the lymphoid gate is represented on the y axis. Testing was done on the same day as the proliferation data was collected. Individuals 18 and 150 were anti-HCV positive but HCV RNA negative at the time of testing.

Since IL-10 is an immunomodulatory cytokine that suppresses TH1 type adaptive immunity, we investigated the relationship between IL-10 induction by HCV proteins and HCV-specific T cell proliferation in 10 HIV/HCV-coinfected individuals. A positive HCV-specific proliferation response (stimulation index greater than or equal to 3) was observed in only one individual (018, Figure 6a, black bar), who demonstrated a positive response to HCV core but not NS3 and NS4. After 16 hour exposure to HCV antigens, 6 of the 10 coinfected individuals tested demonstrated an HCV protein-induced increase in the number of IL-10^+ ^cells (grey bars, Figure 6b). Of note, subject 018 was one of the four individuals who did not have increased numbers of IL-10^+ ^cells following exposure to HCV proteins.

## Discussion

In this study, we demonstrated that HCV core, NS3 and NS4 proteins specifically and selectively induce IL-10 production when incubated for 16 hours with PBMC. The cells producing IL-10 in response to the HCV proteins were identified by flow cytometry as CD14^-^CD36^+ ^with lymphoid light scatter characteristics. We previously reported constitutive ex vivo production of IL-10 by this PBMC subset and that this production rapidly stops in vitro [22]. These cells do not fit into one of the major subpopulations of circulating immune cells, but can be reliably isolated from all healthy individuals tested at a frequency of up to 1%. The ex vivo frequency of IL-10-producing PBMC was generally equivalent in the four distinct groups we studied, indicating that the differences seen after 16 hour culture were caused by differential responses to the HCV proteins. While 16 hour culture with HCV proteins increased the number of IL-10-producing cells in all groups, the frequency of IL-10^+ ^cells was higher in PBMC from HIV-infected individuals and uninfected controls compared to PBMC from HIV/HCV coinfected individuals or HCV-infected individuals. This demonstrates a propensity for HCV-induced IL-10 production by PBMC from uninfected individuals that is exaggerated by HIV infection and attenuated by chronic HCV infection, even in the setting of HIV coinfection.

We used recombinant HCV core, NS3 and NS4 to investigate cytokine production in response to HCV proteins. Although these three proteins have little sequence or structural similarity and their known functions in HCV replication are very different [23,24], we found that, collectively or individually, HCV core, NS3 and NS4 proteins induced IL-10 production in CD14^-^CD36^+ ^PBMC. Others have also reported that recombinant HCV core, NS3 and NS4 proteins all induce IL-10 production [16,25,26]. However, in these studies, monocytes were identified as the responding cells and, in direct contrast to our results, there was a greater effect on monocytes from HCV-infected individuals than on monocytes from uninfected controls [16,27]. These studies utilized monocytes isolated by overnight adherence to plastic, rather than freshly isolated PBMC. Despite varying protocols and different characteristics of the responding cells, the central point is that multiple distinct HCV proteins stimulate IL-10 production by PBMC. There are other examples of redundancy in HCV protein effects as mice transgenic for hepatic HCV structural or non-structural polyproteins develop hepatic steatosis characteristic of chronic HCV infection [28]. Such functional redundancy of viral proteins is not unique to HCV, as three different HIV proteins (nef, tat and vpr) have all been reported to induce IL-10 production by various PBMC subsets [29-33].

In our study, structural and non-structural HCV proteins induced IL-10. Having both structural and non-structural proteins with redundant immunosuppressive functions may be important in sustaining infection. The availability of these proteins in circulation is temporally separated, and not concurrent. Non-structural proteins are only expressed in actively infected cells, while the core protein can be found in plasma at moderately high levels throughout chronic infection [34,35]. Having multiple proteins that induce IL-10 supports production of an immunosuppressive cytokine whether or not HCV replication is upregulated or infectious virions are present.

In parallel with the redundancy in HCV protein-induced IL-10 production, additional HCV escape mechanisms within both the innate and adaptive immune system have been extensively described. Subversion of the eukaryotic anti-viral interferon-inducible system is a potent evasion strategy employed by many viruses, and several HCV proteins suppress host proteins in this system. HCV E2 inactivates PKR [36], and the HCV serine protease NS3/4a causes proteolysis of Toll-IL-1 receptor domain-containing adaptor inducing IFN-β (TRIF) [37], and inhibits phosphorylation of IRF-3, preventing nuclear translocation and IFN-β gene activation [38]. HCV E2 has also been associated with decreased NK cell function and HCV core stabilizes HLA-E expression and subsequently downregulates NK-mediated cytotoxicity [39-41]. HCV core, E1, NS3, and NS4 are all associated with impaired dendritic cell maturation, thus potentially modulating the function of this integral link to adaptive cellular immunity [16,27,42]. HCV core also downregulates in vitro anti-HCV T cell responses [17,43,44]. While the exact mechanism of HCV protein interaction is unclear, IL-10 inhibits dendritic cell maturation, resulting in an immature phenotype similar to that seen after exposure to HCV core [45]. Not only do HCV proteins modulate immune responses, it is clear that more than one protein has evolved to share similar functions. Production of IL-10 by the cell population described in this paper may stunt dendritic cell maturation, and impair development of anti-HCV cellular immunity through this pathway as well as through a direct effect on responding T cells.

The mechanism for IL-10 induction in PBMC by HCV proteins is unknown, but processing of soluble HCV antigens through the endocytic pathway is unnecessary as blocking antigen processing had no effect on constitutive or induced IL-10 production. A higher percentage of cells from HIV-infected individuals than uninfected controls produced IL-10 in response to HCV proteins. This appears unrelated to lower T cell function in HIV infection as depletion of CD3^+ ^PBMC also did not affect constitutive or HCV-induced IL-10 production by the CD14^-^CD36^+ ^PBMC.

The direct correlation between intracellular IL-10 production and intensity of cell surface CD36 expression supports a possible connection between these molecules. Ligands of CD36, a scavenger receptor, include oxidized low density lipoproteins (oxLDL), thrombospondin, collagen types I and IV, apoptotic cells, long chain fatty acids and plasmodium-infected erythrocytes [46-54]. At least 2 of these ligands, thrombospondin and apoptotic cells, have immunosuppressive or anti-inflammatory effects [55,56]. In addition, peroxisomal proliferator associated receptor-γ (PPAR-γ) mediated differentiation of monocytes towards a less inflammatory status is accompanied by up-regulation of CD36, which has been used to clinical advantage in cerebral malaria [57]. While there is circumstantial evidence for a link between CD36 and induction of IL-10, there has been no reported evidence of interactions between HCV or any of its individual components and CD36. HCV associates with low density lipoproteins in circulation, and uses the LDL receptor and CD81 to enter cells [58]. CD36 is not an HCV cellular receptor however, recent studies suggest that HCV and the CD36 ligand oxLDL do interact [59]. HCV proteins may indirectly activate IL-10 production through a CD36 related mechanism.

IL-10 is an important immunomodulatory cytokine, and recent experiments in the LCMV model of chronic viral infection showed that manipulating levels of IL-10 alone was sufficient to dictate LCMV clearance or persistence [60]. However, studies comparing plasma IL-10 levels in HCV-infected and healthy individuals have been less definitive, with some studies describing control levels of serum IL-10 in chronic HCV infection, while other groups describe a marked increase in IL-10 in chronic infection [61-63]. This raises questions about the value of serum IL-10 measurements, as IL-10 has important localized paracrine effects that are not reflected by these generalized IL-10 determinations. As well, none of these studies determine IL-10 levels during acute infection when it may be pivotal in the establishment of chronic infection. We demonstrated that exposure to HCV antigens in chronically infected individuals produced less IL-10 than seen in HCV naïve individuals. Longer term exposure to HCV antigens apparently conditions the CD36^+ ^population of IL-10^+ ^cells, attenuating HCV protein specific IL-10 induction. Thus, IL-10 levels during acute infection may be more relevant to the establishment of persistent infection than to maintaining chronic infection.

The transience of IL-10 responses to HCV proteins may allow emergence of cellular immune responses that are pathological in the context of chronic HCV infection. Several studies indicate a protective role for IL-10 in the immunopathology of HCV infection including a clinical trial of exogenous IL-10 administration, accumulation of IL-10 producing CD5^+ ^B cells in HCV-infected individuals with mild disease and an association between high IL-10 gene polymorphism and decreased rates of HCV-related liver fibrosis [64-67]. The diminution in IL-10 induction seen in chronic HCV after HCV protein exposure appears to parallel, and possibly support, insidiously progressive liver disease.

Coinfection with HIV is an important issue in HCV infection because of its relatively high prevalence, higher HCV virus loads in HIV/HCV coinfected individuals and because of increased HCV-related morbidity in HIV coinfected individuals [68-70]. The higher level of IL-10 induction in PBMC from HIV-infected individuals may allow the establishment of more aggressive HCV infection, with downregulation of anti-HCV responses permitting increased HCV replication and higher HCV virus loads. However, this could depend on the order of HCV and HIV infection, with stronger anti-HCV cellular immunity developing when HCV infection precedes HIV-associated CD8^+ ^T cell activation during the asymptomatic phase of HIV infection. Enhanced CD8^+ ^T cell immunity against HCV in HCV/HIV coinfected individuals is likely to accelerate liver disease, while those who acquire HCV following the generalized decline in cellular immunity associated with progressive HIV infection are likely to have especially weak HCV-specific cellular immunity. Thus, there are 2 distinct groups of HIV/HCV coinfected individuals with respect to HCV-specific cellular immunity and its potential role in viral suppression and in the pathogenesis of liver disease.

Chronic HCV infection mitigates IL-10 production induced by HCV proteins. This is true in the group of three HCV monoinfected individuals, as well as the 10 HIV/HCV coinfected people. Though the monoinfected group is small, the two groups with chronic HCV infection have a total of thirteen individuals, and both have similar results. The limited IL-10 response is even more marked considering that the HIV monoinfected group has the highest IL-10 induction of all groups. Therefore, the lack of IL-10 induction in the setting of chronic infection is likely not an artifact of the small sample size, but a real phenomenon that can even supplant the expected HIV-associated IL-10 induction.

Consistent with previous reports, we found that HCV specific T cell proliferation is rare in chronic HCV infection with HIV coinfection a[71,72]. In fact, the only individual with anti-HCV proliferative responses is also one of the individuals with the least number of IL-10 producing cells after HCV protein exposure.

## Conclusion

We have demonstrated the presence of a novel population of PBMC that act differentially in response to HCV proteins in uninfected and HIV- or HCV-infected individuals. This differential IL-10 response to HCV antigens is consistent with the generation of an immunoregulatory environment that promotes viral persistence in acute exposure while allowing disease progression in the chronic state. The behaviour of this CD36^+^CD14^- ^population offers insight into how HCV establishes viral persistence through decreased cellular immune responses and how this is exacerbated by HIV infection.

## Methods

### Study Participants

HIV-infected and HIV/HCV-coinfected individuals were recruited from the St. John's General Hospital Infectious Disease Clinic, St. John's, NL, Canada. HCV-infected individuals were recruited from the Capital Health Queen Elizabeth Hospital Hepatitis Clinic in Halifax, NS, Canada. Ethics approval for this project was obtained from the Human Investigation Committee at each institution and all subjects provided informed written consent for blood collection and immunological studies. Uninfected individuals were recruited from Memorial University of Newfoundland Faculty of Medicine personnel.

### Identification of HCV infection

HCV exposure was ascertained by testing for serum anti-HCV antibodies using second or third generation EIA assays from Ortho Diagnostics (Mississauga, ON). HIV status was determined by commercially available PCR testing by Abbott Diagnostics (Mississauga, ON).

To demonstrate the presence of HCV RNA, total nucleic acids were extracted from plasma samples, using NucliSens^® ^Lysis Buffer and the Nuclisens^® ^Isolation Kit (Organon Teknika, Durham, NC). Briefly, 200 μL of plasma was added to 1 mL of lysis buffer. Silica beads were added to bind free nucleic acids, followed by successive washes with 70% ethanol, 95% ethanol, and acetone. Nucleic acids were removed from the silica at 56°C with the elution buffer provided. 30 μL of eluant was frozen at -20°C if not used immediately for cDNA synthesis.

Complementary DNA (cDNA) was produced from isolated RNA using a first-strand cDNA synthesis kit (Amersham Biosciences, Baie d'Urfé, Québec). 8 μL of extracted nucleic acid was denatured at 65°C for 5 minutes and added to a reaction mix containing random hexamer primer, deoxyribonucleotides, and Moloney Murine Leukemia Virus reverse transcriptase (MMLV-RT). Reactions were placed at 37°C for 1 hour followed by MMLV-RT inactivation at 95°C for 5 minutes. cDNA was stored at -80°C until needed.

cDNA was amplified by polymerase chain reaction (PCR) with primers specific for the highly conserved HCV 5'-untranslated region. Primers and PCR conditions were modified from Shindo et al. [73] as follows: forward primer 5'-GGCGACACTCCACCATAGATC-3' and reverse primer 5'-GGTGCACGGTCTACGAGACCT-3'. The expected amplicon size was 324 bp. Final reactions included 20 mM TRIS-HCl (pH 8.4), 50 mM KCl, 0.5 mM MgCl_2_, 0.025 U/μL DNA polymerase (Invitrogen, Burlington, Ontario), and 0.5 μM MG18 and MG321 primers (Invitrogen, Burlington, Ontario) in 50 μL. Samples were initially denatured at 95°C for 2 minutes, followed by thirty cycles of amplification (95°C for one minute, 60°C for one minute, and 72°C for one minute). PCR products were analyzed on 2% agarose gels with ethidium bromide visualization and stored at -20°C.

### PBMC isolation and in vitro stimulation

Acid citrate dextrose (ACD) treated whole blood was obtained by venipuncture from each individual and PBMC were isolated by Ficoll-HyPaque Plus (GE Health Care, Baie d'Urfé, PQ, CA) density gradient centrifugation. Cells were washed, counted and suspended at 1 × 10^6^/mL in lymphocyte medium (RPMI supplemented with 10% FCS, 10 mM HEPES, 2 mM L-glutamine, 1% penicillin/streptomycin, and 2 × 10^-5 ^M 2-mercaptoethanol; all from Invitrogen, Burlington, ON, CA).

To determine the effect of HCV proteins on PBMC from healthy and infected individuals, 1.5 × 10^6 ^freshly isolated PBMC were incubated in lymphocyte medium at 37°C for 16 hours with 10 μg/mL Brefeldin A (Sigma Chemical Co., St. Louis, MO, USA) and 2.5 μg/mL recombinant HCV core, NS3, and NS4 proteins (Virogen, Watertown, MA), 2.5 μg/mL recombinant HIV p24 from the same source (Virogen) or 2.5 μg/mL various control proteins. Initially, various concentrations (1, 2.5, 5, and 7.5 μg/mL) were tested in 3 individuals to determine if there was a dose response. All concentrations tested demonstrated results within 0.01% of each other in each individual (data not shown), and 2.5 μg/mL was used in subsequent studies as it was compareable to other studies of HCV proteins in human PBMC. The HCV antigens were expressed in E. coli as β-galactosidase fusion proteins, and supplied in a urea-based buffer, therefore, negative controls included β-galactosidase (Calbiochem, La Jolla, CA) in the same buffer as the viral antigens. Polymixin B sulfate (PMB; Sigma) was added at 100 μg/mL to block any endotoxin-induced cytokine production as described previously[74]. For some experiments, the lysosomal antigen processing pathway inhibitor chloroquine (Sigma) was added at 50 μM to PBMC 30 minutes before addition of HCV antigens to investigate whether antigen processing was involved in IL-10 induction. Cytokine production was assessed by intracellular flow cytometry.

### T cell depletion

For some experiments, CD3^+ ^T cells were removed from fresh PBMC by depletion with magnetic beads. Ten million fresh PBMC were washed and resuspended in cold depletion buffer (PBS supplemented with 5 mM EDTA and 0.5% BSA) at 3 × 10^6^/mL, and a 10:1 bead-to-target cell ratio with mouse anti-human CD3-coated magnetic beads (Dynal ASA, Lake Success, NY). Cells and beads were rotated together for 45 minutes at 4°C, and bead bound CD3^+ ^cells were removed using a magnet. Depletion efficiency was >97% as determined by flow cytometry. The remaining cells were resuspended in lymphocyte medium and exposed to HCV and HIV proteins as described above.

### Flow cytometry

#### Surface staining

PBMC subpopulations were phenotyped by surface and intracellular flow cytometry using the following antibodies: CD3-phycoerythrin (PE; Clone UCHT1, DakoCytomation, Mississauga, ON), CD4-fluorescein isothiocyanate (FITC; Clone RPA-T4, BD Pharmingen, San Diego, CA), CD8-peridinin chlorophyll (PerCP; Clone RPA-T8, BD Pharmingen), CD14-FITC or CD14-PE (Clone M5E2, BD Pharmingen), CD36-PE (Clone CB38, BD Pharmingen), CD19-FITC (Clone HIB19, BD Pharmingen), CD56-FITC (Clone HA58, BD Pharmingen) and CD61-PerCP (clone VI-PL2, BD Pharmingen). To each tube, IL-10 and CD36 were added, along with one other surface marker for each major cell type (e.g. CD36, IL-10, and CD3). Through this strategy, 3 color flow cytometry was used to identify the cell population of interest. All steps were performed at 4°C, and incubations were carried out in the dark to prevent fluorochrome photobleaching. Approximately 5 × 10^5 ^PBMC were washed with flow cytometry buffer (PBS supplemented with 0.1% bovine serum albumin (BSA), 5 mM EDTA and 0.02% sodium azide, all from Sigma), resuspended in 600 μL and incubated for 20 minutes with 0.5 μg antibody against surface antigens or appropriate isotype controls. Cells were washed, fixed with 0.5 mL 1% paraformaldehyde (Sigma) for 20 minutes, resuspended in 250 μL of 1% paraformaldehyde, and stored at 4°C until analysis on a FACScan flow cytometer (Becton Deckinson). CellQuest Pro™ and WinMDI™ were used for data analysis.

#### Intracellular staining

Cells were stained as above for surface antigens with an additional permeabilization step after fixation. Cells were incubated with either 0.5 mL 0.2% saponin (Sigma) in PBS or DAKO permeabilization reagent and anti-IL-10 FITC (clone JES9D7 Caltag, Burlingame, CA), anti-IFN-γ FITC, (clone B27, Caltag), anti-IL-2 FITC, (clone MQ1-17H12 Caltag) anti-IL-12 FITC (clone C8.6 Caltag) antibodies or appropriate isotype controls for 20 minutes at 4°C in the dark. After washing with 3 mL flow buffer, cells were resuspended in 1% paraformaldehyde until analysis.

Total PBMC were incubated for 16 hours with either 5 μg/mL phytohemagglutinin, 10 nM phorbol 12-myristate 13-acetate (PMA)/100 nM ionomycin, or 200 ng/mL lipopolysaccaride to provide positive controls for IFN-γ, IL-2, IL-10, and IL-12 production, respectively. Brefeldin A was added one hour after the various stimulants. Lymphoid cells were gated and stained for CD8 and IFN-γ production, total PBMC for IL-2 production, and monocytes were gated for IL-12 and IL-10 production.

### Proliferation assays

Cellular immune responses were measured by standard 5 day thymidine incorporation assay. PBMC were washed twice with proliferation medium (lymphocyte medium with 10% human AB serum (Atlanta Biologicals, lot number M0102) substituted for FBS) and 1 × 10^5 ^cells/well were incubated in triplicate with either plain medium, 5 μg/mL phytohemagglutinin (ICN Biomedicals Inc., Costa Mesa, CA), 2.5 μg/mL Candida albicans (Greer Laboratories Inc., Lenoir, NC), or 2 μg/mL HCV core, NS3, NS4 or HIV p24 (all Virogen) for 5 days. One μCi of tritiated (^3^H)-thymidine (Perkin Elmer Life Sciences, Boston, MA) was added to each well on day 4, and the assay was harvested onto glass fiber mats 18 hours later using a semi-automated harvester (Tomtec Harvester 96 Mach M III, Hamden, CT). Incorporated ^3^H-thymidine was measured by a 96 well scintillation counter (TopCount, Packard, Meriden, CT). Stimulation indices were calculated as follows:

$$\text{Stimulation Index}=\frac{{\text{cpm}}_{\text{antigen}}}{{\text{cpm}}_{\text{background}}}$$

### Statistical Analyses

All statistical analyses were performed using SPSS version 9 (SPSS Inc., Chicago, IL). In figure 2, the group sizes were small and unequal. Therefore, nonparametric tests were used to compare means. Within a group, the Mann-Whitney U test was used, while the Kruskal-Wallis test was used to test for differences between groups, as there were greater than 3 groups. Neither test depends on the data distribution for calculation, and the Kruskal-Wallis for multiple comparisons adjusts the alpha value to avoid inflation of type I error. In figure 3, the Fisher's exact test was used to compare the categorical data. All graphs indicate mean values and error bars represent standard error of the mean.

## Authors' contributions

LB designed and carried out the experiments, and drafted the manuscript. MGa carried out some of the proliferation assays. CH, MIB, GH, and KP participated in the design of the study and recruitment of patients. MG designed and coordinated the study, and drafted the manuscript.
